# LncRNA MBNL1-AS1 Suppresses Cell Proliferation and Metastasis of Pancreatic Adenocarcinoma through Targeting Carcinogenic miR-301b-3p

**DOI:** 10.1155/2023/6785005

**Published:** 2023-03-01

**Authors:** Chouman Sulidankazha, Alidake, Hai Lin, Tieying He, Wei Han, Qilong Chen

**Affiliations:** ^1^Department of Pancreatic Surgery, Digestive and Vascular Surgery Center, The First Affiliated Hospital of Xinjiang Medical University, Urumqi 830054, Xinjiang Uygur Autonomous Region, China; ^2^Department of Breast Surgery, Digestive and Vascular Surgery Center, The First Affiliated Hospital of Xinjiang Medical University, Urumqi 830054, Xinjiang Uygur Autonomous Region, China

## Abstract

Pancreatic adenocarcinoma (PAAD) has been a huge challenge to public health due to its increasing incidence, frequent early metastasis, and poor outcome. The molecular basis of tumorigenesis and metastasis in PAAD is largely unclear. Here, we identified a novel tumor-suppressor long noncoding RNA (lncRNA) MBNL1-AS1, in PAAD and revealed its downstream mechanism. Quantitative real-time PCR (qRT-PCR) data showed that MBNL1-AS1 expression was significantly downregulated in PAAD tissues and cells, which was closely associated with metastasis and poor prognosis. Cell counting kit-8 (CCK-8) assay, transwell assay, and western blot verified that overexpression of MBNL1-AS1 suppressed cell proliferation, migration, and epithelial mesenchymal transformation (EMT) behavior in PAAD cells. By using a dual luciferase reporter gene system, we confirmed that miR-301b-3p was a direct target of MBNL1-AS1. Further mechanismic study revealed that upregulation of miR-301b-3p abolished the inhibitory effect of MBNL1-AS1 overexpression on cell proliferation, tumorigenesis, migration and EMT. Our results demonstrate that MBNL1-AS1 plays a tumor-suppressive role in PAAD mainly by downregulating miR-301b-3p, providing a novel therapeutic target for PAAD.

## 1. Introduction

Pancreatic adenocarcinoma (PAAD) is a malignant tumor of the digestive tract. Its incidence rate is increasing all over the world, and its prognosis is usually poor [1]. Although the treatment for other cancers has made great progress in recent years, the survival rate of PAAD has been stagnant, and the 5-year survival rate remains between 5% and 10% [2]. It is prone to distant metastasis in the early stage, and there are no specific symptoms and signs in the early stage of PAAD, which poses a huge challenge for the improvement of PAAD prognosis. Understanding the molecular mechanism of pancreatic tumorigenesis and metastasis may help to develop new diagnostic and therapeutic methods.

Long noncoding RNAs (lncRNAs) are considered as tumor regulators and have attracted increasing attention in the field of cancer research over the past decades [3]. LncRNA is defined as a class of noncoding RNAs (ncRNAs) with a length of more than 200 nucleotides [4]. LncRNAs account for a large proportion of the human genome and participate in a set of physiological or pathological process through interacting with DNAs, mRNAs, ncRNAs, and proteins [5]. Numerous studies have showed that lncRNAs are specifically expressed in tumors and regulate multiple tumor biological processes, including sustaining proliferation signaling, evading growth suppressors, enabling replicative immortality, resisting cell death, etc. [6–8]. Among the aberrantly expressed lncRNAs, MBNL1-AS1 is downregulated and regarded as a novel tumor-suppressive lncRNA in several common cancers, like colon cancer, breast cancer, and non-small lung cancer [9–11]. MBNL1-AS1 has been proven to repress cell proliferation and migration in these cancers. In addition, MBNL1-AS1 is reported to be upregulated in acute myocardial infarction, and silencing MBNL1-AS1 reduces myocardial injury in animal models [12]. From the current research results, we know that MBNL1-AS1 reduces cell survival in both cancer and noncancer cells. However, it is still unclear whether and how MBNL1-AS1 plays tumor-suppressive roles in PAAD.

MicroRNAs (miRNAs) are a class of short ncRNAs consisting of about 22 nucleotides. They usually form incomplete base pairs with the 3′-untranslated region (3′-UTR) of the target gene and play a negative role in regulating gene expression [13, 14]. Specific miRNAs have been reported to regulate of a large amount of cellular biological processes, such as cell proliferation, differentiation, migration, epithelial mesenchymal transformation (EMT), etc. [15]. Among them, a lot of functional miRNAs are identified in the cancer progression. For example, miR-106b-5p contributes to the lung metastasis of breast cancer [16]. miR-19a facilitates metastasis and EMT in prostate cancer [17]. miR-331-3p promotes drug resistance in pancreatic cancer [18]. Recently, miR-301b-3p is reported to function as a tumor promoter in breast cancer, lung cancer, and colorectal cancers [19–21]. Interestingly, a few evidences have suggested that miR-301b links with gemcitabine resistance and cell invasion in pancreatic cancer, and miR-301b plays its role in pancreatic cancer through downregulating the expression of tumor-suppressor TP6 [22, 23]. Thus, miR-301b might be an effective therapeutic target for PAAD treatment; however, little is known about its detailed regulatory mechanism in PAAD.

Herein, we detected the expressions of MBNL1-AS1 and miR-301b-3p in PAAD and analyzed the correlation between their expressions and metastasis and the overall survival rate of PAAD patients. Moreover, the role of MBNL1-AS1 and miR-301b-3p in PAAD and their interaction mechanisms were revealed in the present work. The study aims to uncover the molecular mechanism of PAAD development and provide potential targets for the diagnosis and treatment of PAAD.

## 2. Methods and Materials

### 2.1. Clinical Samples

PAAD tissues and the adjacent normal tissues were collected from 52 cases of PAAD patients in the First Affiliated Hospital of Xinjiang Medical University. All the patients received surgical treatment in our hospital from 2019 to 2021 and were diagnosed with PAAD by postoperative pathology. The patients accepted no treatment before the operation, and their clinicopathological data and follow-up data were complete. The surgical resection tissues were stored at −80°C. All the protocols involved with the human tissue samples were in accordance with the World Medical Association Declaration of Helsinki. The study protocol was approved by the ethics committee affiliated with the First Affiliated Hospital of Xinjiang Medical University (approval number: 2021-032), and the study has been granted an exemption from requiring written informed consent.

### 2.2. Cell Culture

HPDE6-C7, Capan-2, AsPC-1, SW1990, PANC-1, and SW1990 cells were purchased from the American Type Culture Collection (ATCC, VA., USA) and cultured in Dulbecco's Modified Eagle Medium (DMEM; Hyclone, UT., USA) supplemented with 10% fetal bovine serum (FBS, Gibco, NY., USA), 100 *μ*g/ml streptomycin, and 100 IU/ml penicillin. The culture medium was kept in an incubator (Liuyi, Beijing, China) with 5% CO2 at 37°C.

### 2.3. Quantitative Real-Time PCR (qRT-PCR)

qRT-PCR was performed to detect the expression of miR-301b-3p and MBNL1-AS1. Briefly, total RNA was first released from cells by using a TRIzol Kit (Invitrogen, CA., USA) according to the manufacturer's protocol. Next, total RNA was reverse- transcribed into cDNA using the PrimeScript RT Reagent Kit (TaKaRa, Dalian, China) or One Step PrimeScript® miRNA cDNA Synthesis Kit (TaKaRa). Finally, qPCR was performed using SYBR qPCR reagents (Takara) and PCR operating instrument (BioRad, CA., USA). Sequences of primers were listed as follows: MBNL1-AS1 (forward:5′-TGGATAAGACAGTCCCTACA-3′, reverse: 5′- ATTGGATTGCTTCCCACATA-3′); GAPDH (forward: 5′-TCAAGGCTGAGAACGGGAAG-3′, reverse: 5′-TGGACTCCACGACGTACTCA-3′) as the internal reference of MBNL1-AS1; miR-301b-3p (forward: 5′- CAGTGCTCTGACGAGGTTG-3, reverse: 5′-TGTCCCAGATGCTTTGACA-3′); U6 (forward: 5′-CTCGCTTCGGCAGCACATA-3′, reverse: 5′-AACGATTCACGAATTTGCGT-3′) as the internal reference of miR-301b-3p. The expression was calculated using the 2^−ΔΔCt^ method.

### 2.4. Western Blot Analysis

Total protein was obtained by RIPA lysis buffer (Solarbio, Beijing, China), and the concentration was measured by a BCA assay kit (Yeasen, Shanghai, China). Then 50 *μ*g protein samples was separated on a sodium dodecyl sulphate-polyacrylamide gel electrophoresis (SDS-PAGE, Beyotime, Shanghai, China) and transferred to polyvinylidene fluoride (PVDF) membranes (Millipore, MD., USA). The membranes were blocked with 5% nonfat milk for 1.5 h and incubated with anti-E-cadherin, N-cadherin, and Vimentin at 4°C overnight. The next day, the membranes were washed and incubated with horseradish peroxidase-labeled IgG antibody at room temperature for 1 h. Images of blots were captured by using the gel imaging system (Bio-Rad, CA., USA), and the original blots are presented in Supplementary file 1.

### 2.5. Cell Transfection

pcDNA-MBNL1-AS1, miR-301b-3p inhibitor, miR-301b-3p mimic, and its negative control (NC) were purchased from Ribo Bio Co., LTD (Guangzhou, China). PANC-1 and SW1990 cells were seeded on a 96-well plate and cultured for 12 h. Then cells were transfected with pcDNA-MBNL1-AS1, miR-301b-3p inhibitor, miR-301b-3p mimic, or NC by using Lipofectamine 2000 reagent (Invitrogen, CA, USA) according to the manufacturer's protocol.

### 2.6. Dual Luciferase Reporter Gene System

According to the binding site between miR-301b-3p and MBNL1-AS1 analyzed by bioinformatic tools, the wild-type sequence of MBNL1-AS1 and the mutant sequence of MBNL1-AS1 were designed, synthesized, and inserted into the vector pRL-TK (Promega, Beijing, China). PANC-1 and SW1990 cells were seeded on a 96-well plate and transfected with the reconstructed vector containing MBNL1-AS1 sequences, miR-301b-3p mimic, or NC mimic, by using Lipofectamine 2000 regents (Invitrogen, CA., USA). 48 h later, the cells were lysed for measurement of luciferase activity.

### 2.7. Cell Counting Kit (CCK-8) Assay

PANC-1 and SW1990 cells (2 × 10^3^ cells/well) were seeded in 96-well plates, and CCK-8 solution (10 *μ*mol/L) was added into each well at different timepoint (0 h, 24 h, 48 h, and 96 h). After incubating for 2 h at 37°C, the absorbance (OD value) of cells at 450 nm was measured with a microplate reader.

### 2.8. Apoptosis

Cell apoptosis was detected using the AnnexinV-FITC/PI Apoptosis Detection Kit (BD, NJ, USA). Briefly, cells were digested with 0.25% pancreatin, washed with precooled PBS, and centrifuged at 2000 rpm for 10 min. Cell precipitation was suspended with 300 *μ*L binding buffer, mixed evenly with 5 *μ*L Annexin V-FITC and incubated at room temperature for 15 min in the dark. 5 *μ*L PI was added into cell mixture 5 min before measurement, and 200 *μ*L binding buffer was added into them immediately before measurement. Flow cytometry was utilized for the measurement of the apoptotic rate.

### 2.9. Transwell Assay

100 *μ*L PANC-1 or SW1990 cells (5 × 10^4^ cells/well) were seeded in the upper chambers of a 24-well Transwell chamber (BD, NJ, USA), while 500 *μ*L DMEM supplemented with 10% FBS was added to the lower chamber. After being cultured for 48 h, migrated cells in the lower chamber were fixed with 4% paraformaldehyde for 15 min and stained with 0.1% crystal violet (Sigma, MO., USA) for 15 min at room temperature. After being washed and dried, cells were photographed under a microscope (Olympus, Tokyo, Japan).

### 2.10. Xenograft Tumors

PANC-1 cells in the logarithmic growth phase were digested with 0.25% trypsin and resuspended in PBS after washing. 200 *μ*L cell suspension (1 × 10^6^ cells) was subcutaneously injected into the right armpit of nude mice using a 1 mL syringe. Tumor growth was recorded within 28 d after injection. The mice were sacrificed at 28 day and tumors were weighed.

### 2.11. Statistical Analysis

GraphPad Prism 8.0 (GraphPad Software, CA., USA) was used for data analysis and graphics drawing. Each experiment was independently repeated at least 3 times, and all data were presented as the mean ± standard deviation. Data in two groups was compared by using Student's *t*-test, and *p* < 0.05 was considered statistically significant.

## 3. Results

### 3.1. MBNL1-AS1 Was Downregulated in PAAD and Closely Related to PAAD Progression

**Figure 1:**
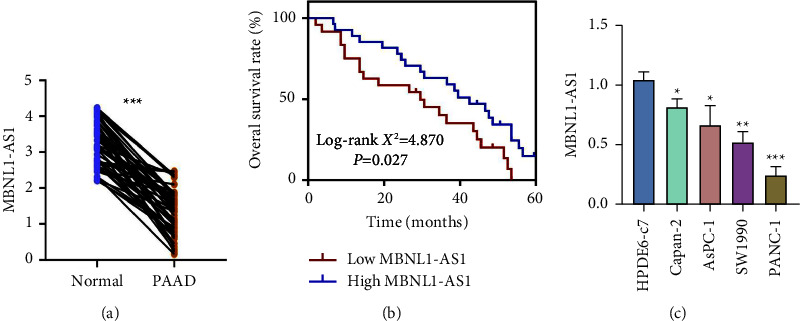
MBNL1-AS1 was downregulated in PAAD and closely related to PAAD progression: (a) MBNL1-AS1 expression in PAAD tumor tissues and adjacent normal tissues. (b) The overall survival rate of PAAD patients with high MBNL1-AS1 expression or low MBNL1-AS1 expression. (c) MBNL1-AS1 expression in the normal pancreatic ductal epithelial cells (HPDE6-C7) and PAAD cell lines (capan-2, AsPC-1, SW1990, and PANC-1). ^*∗*^*P* < 0.05, ^*∗∗*^*P* < 0.01, and ^*∗∗∗*^*P* < 0.001.

We first collected 52 cases of surgical resection tissues from PAAD patients and detected the expression of MBNL1-AS1 in PAAD tumor tissues and adjacent normal tissues. The qRT-PCR data in Figure 1(a) indicated that the MBNL1-AS1 expression of tumor tissues was lower than that of normal tissues. Then these tumor tissues were divided into two groups according to the average value of MBNL1-AS1 expression, 28 cases with high MBNL1-AS1 levels and 24 cases with low MBNL1-AS1 levels, and the relationship between MBNL1-AS1 and their clinicopathological features was shown in Table 1. Low expression of MBNL1-AS1 was significantly associated with poor differentiation degree, high TNM stage, and lymph node metastasis, while there was no significant correlation between MBNL1-AS1 and other variables, including age, gender, tumor location, or alcohol history (Table 1). Additionally, Kaplan–Meier analysis data indicated that lower MBNL1-AS1 expression was associated with a poorer prognosis and shorter overall survival of PAAD (Figure 1(b)). Meanwhile, the MBNL1-AS1 expression in PAAD cell lines (Capan-2, AsPC-1, SW1990, and PANC-1) was lower than that in normal pancreatic ductal epithelial cells (Figure 1(c)). Taken together, the data suggested that MBNL1-AS1 was downregulated in PAAD and closely related to PAAD progression and metastasis.

### 3.2. Overexpression of MBNL1-AS1 Suppressed Cell Proliferation, Migration, and EMT in PAAD

**Figure 2:**
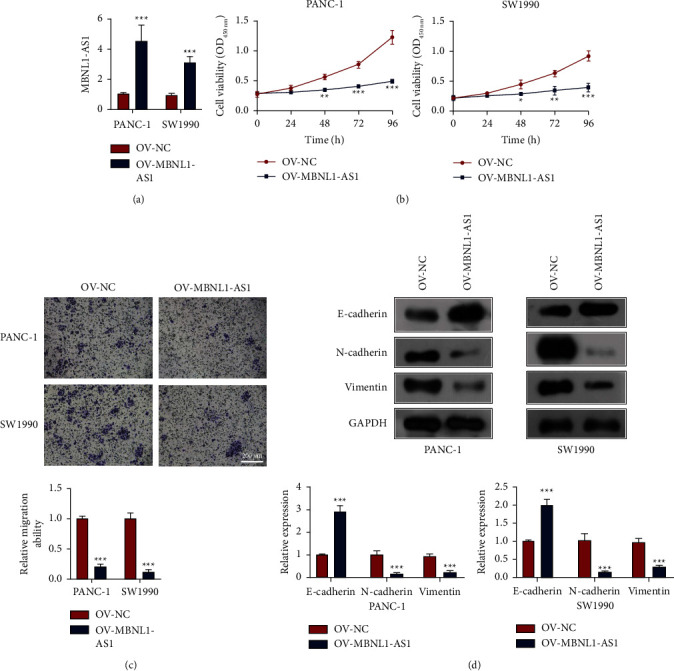
Overexpression of MBNL1-AS1 suppressed cell proliferation, migration, and EMT in PAAD. (a) Verification of effect of transfection with pcDNA-MBNL1-AS1 in PANC-1 and SW1990. (b) Cell viability of PANC-1 and SW1990 transfected with or without pcDNA-MBNL1-AS1 at different time. (c) Cell migration of PANC-1 and SW1990 transfected with or without pcDNA-MBNL1-AS1. Scale bar = 200 *μ*m. (d) The EMT markers (E-cadherin, N-cadherin, and vimentin) in PANC-1 and SW1990 transfected with or without pcDNA-MBNL1-AS1. OV, means overexpression. Compared to the OV-NC group, ^*∗*^*P* < 0.05, ^*∗∗*^*P* < 0.01, and ^*∗∗∗*^*P* < 0.001.

Due to the significance of abnormal expression in SW1990 and PANC-1, the two cell lines were chosen for the following experiments. To investigate the role of MBNL1-AS1 in PAAD, pcDNA-MBNL1-AS1 was transfected into SW1990 and PANC-1 for overexpressing MBNL1-AS1 (Figure 2(a)). Then, CCK-8 assay was performed to evaluate cell proliferation, and the results in Figure 2(b) showed that overexpression of MBNL1-AS1 suppressed the cell viability of the PAAD cells. Next, transwell assay was used to evaluate cell migration, and we found that cell migration ability was also inhibited by overexpression of MBNL1-AS1 (Figure 2(c)). Finally, the expression of EMT markers was measured through western blot (Figure 2(d)). The data indicated that MBNL1-AS1 overexpression elevated the expression of an epithelial marker (E-cadherin) but decreased the expression of mesenchymal marker (N-cadherin and Vimentin), suggesting that MBNL1-AS1 hindered EMT behavior in PAAD cells. Collectively, these results demonstrated that upregulating MBNL1-AS1 suppressed proliferation, migration and EMT in PAAD cells.

### 3.3. MiR-301b-3p was Targeted by MBNL1-AS1 in PAAD

**Figure 3:**
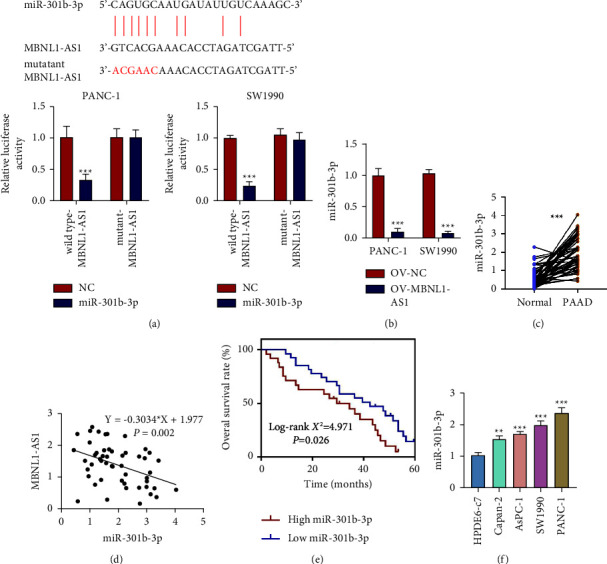
MiR-301b-3p was targeted by MBNL1-AS1 in PAAD. (a) Dual luciferase reporter gene assay was used to verify the targeting relationship between miR-301b-3p and MBNL1-AS1. (b) The effect of pcDNA-MBNL1-AS1 on the miR-301b-3p expression in PANC-1 and SW1990. (c) The miR-301b-3p expression in adjacent normal tissues and PAAD tumor tissues. (d) Linear regression analysis of the relationship between miR-301b-3p and MBNL1-AS1 expression. (e) The overall survival rate of PAAD patients with high miR-301b-3p expression or low miR-301b-3p expression. (f) The miR-301b-3p expression in different cell lines. ^*∗*^*P* < 0.05, ^*∗∗*^*P* < 0.01, and ^*∗∗∗*^*P* < 0.001.

Through bioinformatics analysis, we found that miR-301b-3p could bind to MBNL1-AS1 theoretically, which was verified by the next dual luciferase reporter gene system (Figure 3(a)). We found that miR-301b-3p mimic reduced the luciferase activity in PAAD cells transfected with the wild-type sequence of MBNL1-AS1, rather than that in cells transfected with the mutant sequence of MBNL1-AS1. And miR-301b-3p expression in PANC-1 and SW1990 could be reduced by overexpressing MBNL1-AS1 (Figure 3(b)).

Subsequently, we detected miR-301b-3p expression in PAAD tissues and normal tissues. As expected, miR-301b-3p was upregulated in PAAD tissues (Figure 3(c)) and was negatively related to MBNL1-AS1 expression (Figure 3(d)). Likewise, we categorized these tumor tissues into a miR-301b-3p high-expression group and a miR-301b-3p low-expression group according to the average level of miR-301b-3p. Contrary to MBNL1-AS1, miR-301b-3p expression was closely related to differentiated degree, TNM stage, and lymph node metastasis (Table 2). Moreover, Kaplan–Meier analysis illustrated that the patients with higher miR-301b-3p expression had a poorer prognosis (Figure 3(e)). In addition, the miR-301b-3p expression in PAAD cell lines was shown to be higher than that in normal pancreatic ductal epithelial cells (Figure 3(f)). Altogether, these data miR-301b-3p was a target of MBNL1-AS1 and involved in the PAAD progression.

### 3.4. MBNL1-AS1 Suppressed Cell Proliferation and Metastasis in PAAD by Targeting miR-301b-3p

**Figure 4:**
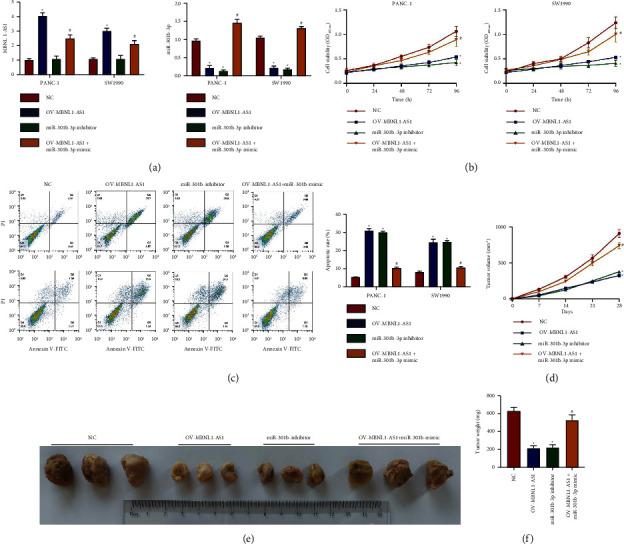
MBNL1-AS1 suppressed cell proliferation in PAAD by targeting miR-301b-3p. (a) Verification of effect of transfection with miR-301b-3p inhibitor or miR-301b-3p mimic and pcDNA-MBNL1-AS1 in PANC-1 and SW1990 with different intervention. (b) Cell viability in PANC-1 and SW1990 with different intervention at different time. (c) Cell apoptosis in PANC-1 and SW1990 with different intervention. (d–f), The stably transfected PANC-1 cells were subcutaneously into nude mice, tumor growth was recorded within 28 days after transplantation (d). The mice were sacrificed at 28 days and tumors were weighed (e-f). Compared to the NC group, ^*∗*^*P* < 0.05. Compared to the OV-MBNL1-AS1 group, ^#^*P* < 0.05.

To verify whether MBNL1-AS1 suppressed PAAD malignancy by targeting miR-301b-3p, the function recovery experiment was conducted by downregulating miR-301b-3p alone or simultaneously overexpressing miR-301b-3p and MBNL1-AS1 in PANC-1 and SW1990 cells (Figure 4(a)). As shown in Figures 4(b) and 4(c), both MBNL1-AS1 restoration and miR-301b-3p depletion reduced cell viability but promoted cell apoptosis in PANC-1 and SW1990 cells, which were reversed by simultaneously overexpressing miR-301b-3p and MBNL1-AS1. Through the tumorigenesis experiment in vivo, we found that either upregulating MBNL1-AS1 or inhibiting miR-301b-3p restrained tumor growth in vivo, while miR-301b-3p mimic abolished the tumor-suppressive effect of MBNL1-AS1 upregulation (Figures 4(d)–4(f)).

**Figure 5:**
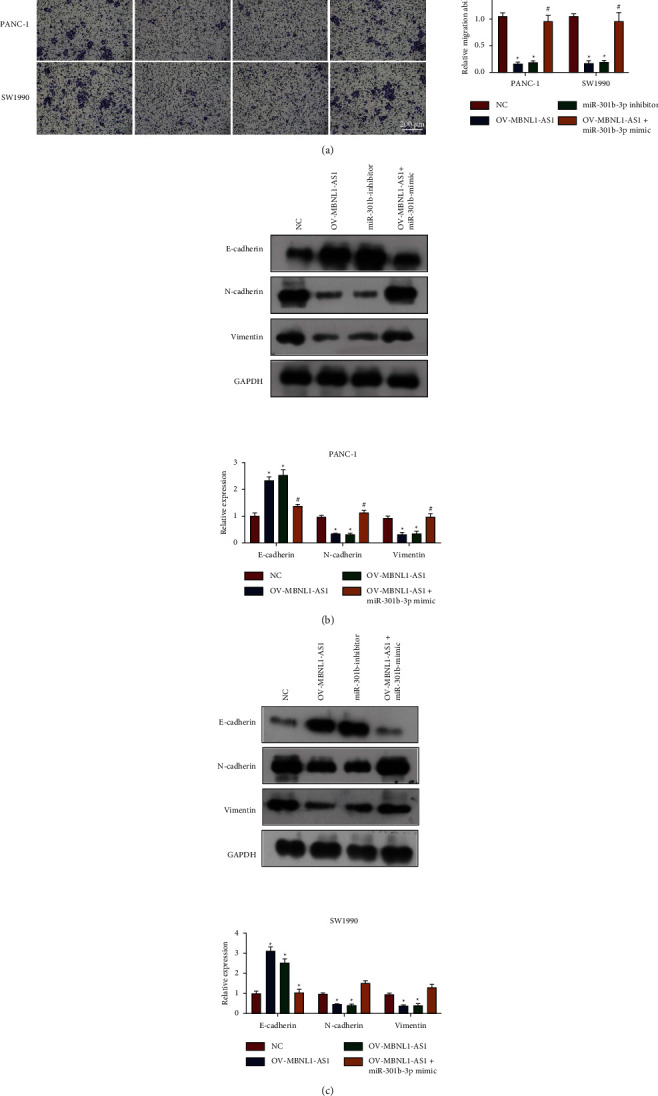
MBNL1-AS1 suppressed cell metastasis in PAAD by targeting miR-301b-3p. (a) Cell migration ability of PANC-1 and SW1990 with different intervention. Scale bar = 200 *μ*m. (b and c), The EMT markers (E-cadherin, N-cadherin and vimentin) in PANC-1 and SW1990 with different intervention. OV means overexpression. Compared to the NC group, ^*∗*^*P* < 0.05. Compared to the OV-MBNL1-AS1 group, ^#^*P* < 0.05.

Additionally, both MBNL1-AS1 upregulation and miR-301b-3p downregulation reduced cell migration ability and EMT behavior in PANC-1 and SW1990 cells (Figures 5(a)–5(c)). Nevertheless, overexpressing miR-301b-3p and MBNL1-AS1 simultaneously counteracted the suppressive effect of MBNL1-AS1 restoration on those aggressive processes in PAAD cells.

To sum up, it is suggested that MBNL1-AS1 hindered PAAD development partly by targeting the tumor-promoting miR-301b-3p.

## 4. Discussion

In recent years, PAAD has attracted increasing attention for its growing incidence and high mortality [24, 25]. Owing to the rapid growth of PAAD cells, abundant blood vessels and lymphatic vessels around the pancreas, and the incomplete envelope of the pancreas itself, PAAD is prone to metastasis at the early stage [26, 27]. EMT, a transition that epithelial cells lose polarity and connections between cells, acquire the ability of infiltration and migration, and become cells with interstitial morphology and characteristics, is the main driving force of migration [28]. In this study, we explored the driving molecular of cell proliferation, migration, and EMT behavior in PAAD, hoping to improve the understanding of the PAAD development.

Accumulating evidence showed the dysregulation of specific ncRNAs could be observed in almost all cancers, and some of these ncRNAs play critical roles in the cancer development and progression [29]. Over the past few decades, a large number of ncRNAs (including lncRNAs and miRNAs) have been recognized as cancer-associated regulators [30–33]. The present work firstly showed that MBNL1-AS1 expression was significantly downregulated in PAAD tissues and cells and demonstrated that overexpression of MBNL1-AS1 suppressed cell proliferation, migration, and EMT behavior in PANC-1 and SW1990 cells. MBNL1-AS1 is a novel lncRNA discovered in recent years. Previous studies have proved that MBNL1-AS1 plays a tumor-suppressive role in several types of cancers, such as breast cancer, lung cancer, and bladder cancer [34–36]. The role of MBNL1-AS1 in PAAD has not been clarified until our study.

According to the competitive endogenous RNA (ceRNA) hypothesis, lncRNAs exert their biology roles usually by sponging miRNAs to regulate genes expression [37]. To date, some miRNAs have been verified to be the target of MBNL1-AS1, including miR-135a-5p in lung cancer and bladder cancer, miR-412-3p in colon cancer, and miR-338-5p in retinoblastoma [9, 34, 36, 38]. In the present study, miR-301b-3p was demonstrated to be the target molecular of MBNL1-AS1, and its expression was elevated in PAAD tissues and cells. As previously reported, miR-301b-3p is essential for the initiation of diverse common cancers, including PAAD [22, 39–41]. MiR-301b-3p promotes cell proliferation and migration by inhibiting a lot of downstream effector genes, which function as tumor suppressors in cells [21, 42–44]. Finally, through a function recovery experiment, we verified that upregulation of miR-301b-3p abolished the inhibitory effect of MBNL1-AS1 overexpression on cell proliferation, apoptosis, tumorgenesis, migration, and EMT. These results preliminarily illustrated the underlying regulatory mechanism of PAAD tumorgenesis and metastasis. However, our present work only illustrated the interaction of MBNL1-AS1 and miR-301b-3p in PAAD because of the limited time and conditions. Actually, many miRNAs could be targeted by MBNL1-AS1. Therefore, further studies are needed to screen other functional miRNAs and improve the molecular mechanisms in the future.

## 5. Conclusion

In conclusion, we first identified MBNL1-AS1 as a tumor-suppressive lncRNA in PAAD, and MBNL1-AS1 reduces cell proliferation, migration, and EMT behavior of PAAD cells via sponging and downregulating miR-301b-3p. The findings shed light on a significant role of ncRNAs in the PAAD progression and might provide a theoretical basis for the development of PAAD therapies.

## Figures and Tables

**Table 1:** Relationship between MBNL1-AS1 and clinicopathological characteristics of PAAD.

Characteristics	*N*	(%)	MBNL1-AS1		*χ* ^2^	*P*
			Low (*n* = 24)	High (*n* = 28)		
Age					1.784	0.182
<65	23	44.23	13	10		
≥65	29	55.77	11	18		
Gender					0.119	0.730
Man	29	55.77	14	15		
Female	23	44.23	10	13		
Tumor location					0.197	0.657
Head	33	63.46	16	17		
Body and tail	19	36.54	8	11		
Differentiated degree					5.175	0.023
Well	24	46.15	7	17		
Moderate-poor	28	53.85	17	11		
TNM stage					4.924	0.026
I-II	38	73.08	14	24		
III-IV	14	26.92	10	4		
Lymph node metastasis					9.095	0.003
Negative	29	55.77	8	21		
Positive	23	44.23	16	7		
Alcohol history					0.550	0.458
Negative	21	40.38	11	10		
Positive	31	59.62	13	18		

**Table 2:** Relationship between miR-301b-3p and clinicopathological characteristics of PAAD.

Characteristics	*N*	(%)	miR-301b-3p		*χ* ^2^	*P*
			Low (*n* = 27)	High (*n* = 25)		
Age					0.349	0.554
<65	23	44.23	13	10		
≥65	29	55.77	14	15		
Gender					1.322	0.250
Man	29	55.77	13	16		
Female	23	44.23	14	9		
Tumor location					1.514	0.219
Head	33	63.46	15	18		
Body and tail	19	36.54	12	7		
Differentiated degree					6.385	0.012
Well	24	46.15	17	7		
Moderate/poor	28	53.85	10	18		
TNM stage					7.137	0.008
I-II	38	73.08	24	14		
III-IV	14	26.92	3	11		
Lymph node metastasis					7.629	0.006
Negative	29	55.77	20	9		
Positive	23	44.23	7	16		
Alcohol history					1.406	0.236
Negative	21	40.38	13	8		
Positive	31	59.62	14	17		

## Data Availability

The data used in this study are all included in this paper. The original data are available from the corresponding author upon reasonable request.

## Supplementary Materials

Supplementary Materials Supplementary file 1: Original western blots: A-B, The original western blots of E-cadherin, N-cadherin, Vimentin, and GAPDH in PANC1 cells (A) and SW1990 cells (B) transfected with or without pcDNA-MBNL1-AS1. C-D, The original western blots of E-cadherin, N-cadherin, Vimentin, and GAPDH in PANC1 cells (C) and SW1990 cells (D) with different intervention.
