# Supplementary figures and images for: LncRNA MBNL1-AS1 Suppresses Cell Proliferation and Metastasis of Pancreatic Adenocarcinoma through Targeting Carcinogenic miR-301b-3p
Source: Genet Res (Camb). 2023 Mar 1;2023:6785005. doi: 10.1155/2023/6785005 (PMC9995204; doi:10.1155/2023/6785005)

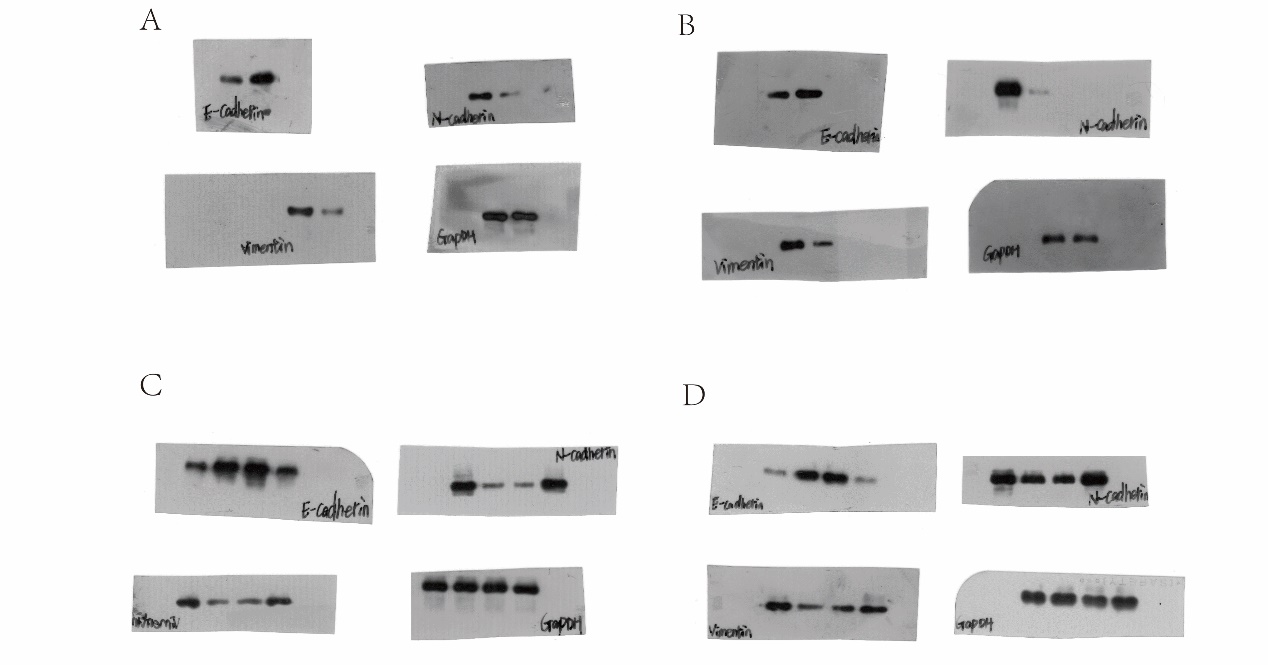

Supplement: Supplementary Materials — Supplementary file 1: Original western blots: A-B, The original western blots of E-cadherin, N-cadherin, Vimentin, and GAPDH in PANC1 cells (A) and SW1990 cells (B) transfected with or without pcDNA-MBNL1-AS1. C-D, The original western blots of E-cadherin, N-cadherin, Vimentin, and GAPDH in PANC1 cells (C) and SW1990 cells (D) with different intervention. [file 6785005.f1.docx]
